# Case-Control Study of Risk Factors and Self-Care Behaviors of Foot Ulceration in Diabetic Patients Attending Primary Healthcare Services in Palestine

**DOI:** 10.1155/2020/7624267

**Published:** 2020-07-22

**Authors:** Basma S. Salameh, Jihad Abdallah, Ehab O. Naerat

**Affiliations:** ^1^Department of Nursing, Arab American University, Jenin, P.O. Box 240, State of Palestine; ^2^Department of Animal Production and Health, An-Najah National University, Nablus P.O. Box 7, State of Palestine; ^3^Department of Diabetic Clinics, Ministry of Health, Jenin, State of Palestine

## Abstract

The purpose of this study is to identify certain sociodemographic, lifestyle, self-care, and foot examination factors that predict the development of diabetic foot ulcers in Palestine. A case-control study was performed in Palestine in 2019. The control group consisted of diabetic patients without foot ulceration (NFU). The case group included diabetic patients who had foot ulcers (DFU) with a size not less than 0.5 cm^2^. The sample of patients was taken from primary healthcare diabetic clinics in Palestine. Findings of the study showed several independent risk factors for developing DFUs, which were smoking, sensory loss to vibration, sensory loss to monofilament, loss of pedal pulse, presence of calluses, nephropathy, retinopathy, and neuropathy. Also, this study has shown that illiteracy and low income were significantly associated with DFU development. Moreover, the current study demonstrated that poor self-care behaviors were associated with DFU. The information gained from the study will contribute to raising awareness and improving health education for diabetic patients and their families with the aim of reducing the complications of diabetes.

## 1. Introduction

The American Diabetes Association defines diabetes mellitus as “a group of metabolic diseases characterized by hyperglycemia resulting from defects in insulin secretion, insulin action, or both. The chronic hyperglycemia of diabetes is associated with long-term damage, dysfunction, and failure of various organs, especially the eyes, kidneys, nerves, heart, and blood vessels” [1].

In the year 2019, statistics were gathered in Palestine regarding the prevalence of diabetes. The number of diabetic patients who have registered in diabetic clinics reached 2503. Females made up 57.4%, while males made up 42.6%. The percentage of patients who were diagnosed at the age of 35 and older was 91.5%. Type I diabetic patients who are dependent on insulin made up 4.2%. Type II diabetic patients depending on oral drugs made up 68.1%. Type II diabetic patients depending on insulin made up 20.8%. The patients who depended on both oral drugs and insulin made up 15% while 0.4% use a diet as a treatment regimen [2]. The prevalence of diabetes in Palestine is increasing tremendously. Diabetes affected 9.7% of the population in the year 2000. In the year 2010, the figure had risen to 15.3%. Diabetes is expected to affect 23.4% of the population by the year 2030 [3].

A number of studies have been conducted to test the relationship between different factors (sociodemographic, lifestyle, mental condition, and foot examination results) and the development of diabetic foot and leg ulcers [4–10]. Although similar studies have been done in the Middle East region, mostly in Saudi Arabia [7, 8, 10], the current study differs in that it is a case-control study, it has been conducted in Palestine (which has different demographics, environment, and lifestyles than Saudi Arabia), and it has included self-care behavior into the analysis. Due to the effect of diabetic foot ulcers on the physical and emotional life of diabetic patients, it is vital to identify the factors that influence the development of diabetic foot ulcers. Thereafter, steps may be taken to reduce the risk of developing diabetic foot ulcers. The purpose of this study is to identify certain sociodemographic factors, life-style factors, and foot examination factors that predict the development of diabetic foot ulcers. The study also aims to determine the effect of self-care behaviors on the development of diabetic foot ulcers.

## 2. Material and Methods

### 2.1. Participants, Study Design, and Sample Size

A case-control design was used to carry out this study. The study was conducted during the period from September 2017 to February 2019. The participants of this study were Type I and Type II diabetic patients attending primary healthcare clinics in Palestine (participants were included consecutively in the clinics). Initially, the minimal required sample size was determined using the Epitools-Epidemiological Calculators site (Sergeant, ESG, 2018, Ausvet; available at: http://epitools.ausvet.com.au) [11]. This utility calculates the sample size required for a case-control study, with specified levels of confidence and power and case and control groups of equal size. A total sample size of 348 (174 per group) was determined assuming a confidence level of 95%, power of 0.80, assumed odds ratio of 3.0, and expected proportion of exposed in controls of at least 0.05 (higher proportions exposed result in smaller samples). The actual sample (total of 413 diabetic patients) consisted of 208 cases (99 males and 109 females) and 205 controls (107 males and 98 females). The control group consisted of diabetic patients without foot ulceration. The cases group included diabetic patients who had foot ulcers with a size not less than 0.5 cm^2^. In addition, the participants must have had a Wagner's scale of 2 (ulcers extend into the tendon, bone, or capsule) or 3 (deep ulcers with osteomyelitis, or abscess leg or foot ulcers). Note that patients with uncomplicated ulcers were not included in the study considering that most diabetic foot patients in Palestine do not seek medical care for uncomplicated foot ulcers due to a lack of awareness or economic hardship.

The ulcer duration must have been more than one week and less than six months at the time of participation in the study. Participants with cognitive impairment or with amputation were not included in the study. It must be taken into consideration that the correct protocol of care for ulcerated patients (such as offloading) is not fully adhered to in Palestine; there is no management by a multidisciplinary team specialized in diabetic foot, and treatment is mostly provided by orthopedic doctors or general practice physicians. Nonulcerated patients were classified according to risk and were recommended to be periodically reviewed for hyperkeratosis debridement; the recommendation was given to the medical team and the nonulcerated patients. In terms of therapeutic shoes and insoles for this group, there are no specialists in this field in Palestine yet.

### 2.2. Instruments

#### 2.2.1. Interview Questionnaire Form

This form is developed by the researchers based on relevant literature in order to collect the necessary information about the study subjects and risk factors. It consisted of the following parts:

- Basic characteristics of participants: age, gender, region, marital status, educational level (illiterate, primary level, secondary level, or university), occupation (unemployed, student, civil servant, self-employed, or other), monthly income, type of diabetes, type of insulin treatment (insulin (how many injections per day), pills, or both), and amount of insulin
- Lifestyle and foot examination factors: cigarette smoking (currently a smoker, an ex-smoker, or a nonsmoker), duration of diabetes, BMI, hypertension (those with *SBP* ≥ 140 *mmHg* or *DBP* ≥ 90 *mmHg* and treat to a goal *SBP* < 140 *mmHg* and goal *DBP* < 90 *mmHg*, or on hypertensive-lowering medications [12]), callus of the feet and foot deformity, and foot skin texture (smooth/moist or dry/cracked)
- Physical assessment (vibration sense and pressure sensation), lab sheet results (hemoglobin A1c test results and fasting blood sugar), and medical records (retinopathy, nephropathy, and neuropathy)

#### 2.2.2. Diabetes Foot Self-Care Behavior Scale (DFSBS)

The DFSBS was used to measure the self-care behavior of diabetes patients in the study. The scale contains 7 items divided into two parts (Table 1) [13].

In the first part (referred to herein as the A subscale), the responses are rated on the number of days patients performed a certain behavior over the last seven days. In the second part (the B subscale), the responses are rated by how often the patients performed a certain behavior in general (1 = never to 5 = always). The number of days for each foot-care measure in the first part was categorized across 5 groups (0 days, 1-2 days, 3-4 days, 5-6 days, and 7 days). Thus, all items on the scale were rated on a 5-point Likert scale where higher scores represent better foot self-care behaviors. The total score was calculated for the whole DFSBS and the two subscales. The total DFSBS score ranged from 7 to 35. Chin and Huang reported a Cronbach's Alpha reliability of 0.75 for the DFSBS [13]. In our study, Cronbach's Alpha was 0.83 for the whole DFSBS, 0.84 for the A subscale, and 0.76 for the B subscale.

### 2.3. Procedure

The consent form and questionnaire were translated into Arabic and, in order to improve content validity, were evaluated by three experts in the field who work in primary diabetic centers (1 physician and 2 nurses). The Arabic version of the DFSBS was piloted on 10 diabetic patients, and necessary changes were made according to the feedback from the pilot sample.

Questionnaires were distributed to diabetic patients attending governmental primary healthcare clinics. Enough time was given to complete both the consent form and the questionnaire. The consent form and questionnaire were then collected. Thereafter, the participants were assessed for neuropathy. A 128 Hz tuning fork was used to assess for vibration sense (over the halluces). Pressure sensation was assessed by applying the SW monofilament at ten sites (9 sites on the plantar surface of the foot; first toe, third toe, fifth toe, first metatarsal head, third metatarsal head, fifth metatarsal head, medial mid foot, lateral mid foot, and heel, and 1 site on the dorsal surface between the base of the first and the second toe). Position sensation was assessed by moving the first toe 10° at the interphalangeal joint. The first toe was moved dorsally and ventrally while holding either side gently with the investigator's thumb and index finger. Medical records were consulted to gather information about the existence of retinopathy, nephropathy, and neuropathy.

### 2.4. Ethical Consideration

The study was approved by the Palestinian Ministry of Health and Helsinki Committee. The approval number is PHRC/HC/167/16. A consent form was given to each participant. The research study was explained, and consent was taken. All information remained confidential. The consent form was not attached to the questionnaire in order to preserve confidentiality.

### 2.5. Statistical Analysis

The data were analyzed in SPSS software v21.0. Basic descriptive statistics (means, frequencies, etc.) were obtained for all participants by group (NFU and DFU). Fisher's Exact Test was used to test differences in frequencies of levels of categorical variables (gender, type of diabetes, etc.) between the NFU and DFU groups while an independent sample *t*-test was used to test for differences between the two groups in means of continuous variables (e.g., age and duration). Differences were declared significant at the 0.05 level (i.e., when *P* < 0.05). Odds ratios of the DFU group compared to the control group were obtained for two-level variables using cross-tabulations.

## 3. Results

### 3.1. Basic Medical and Sociodemographic Characteristics

No differences were found between the DFU and NFU groups in terms of age, gender, marital status, type of diabetes, fasting blood sugar, amount of insulin, or BMI. Significant differences were found in the distribution of patients according to region, educational level, employment status, income, type of medication, HbA1c level, and duration of diabetes.  Table 2 depicts the full results.

### 3.2. Risk Factors

The study assessed associations between smoking, foot examination factors, microangiopathic complications, and foot ulceration. All factors were found to have a significant relationship to foot ulceration except for the existence of dry or cracked foot skin.  Table 3 presents the proportion of participants that have each of these factors by foot ulceration status. The odds ratio and *P* values for each factor are presented as well.

### 3.3. DFSBS

The mean scores of the DFSBS for the DFU and NFU groups are presented in Table 4. The NFU group had significantly higher mean DFSBS scores than the DFU group (*P* < 0.001).

## 4. Discussion

This study is the first of its kind in Palestine to investigate the association of diabetic foot risk factors with the future development of diabetic foot ulcers among adult Palestinian populations and its correlation with self-care behaviors. This study found that region, educational level, and employment had a significant relationship with foot ulceration. Illiterate patients were more common among the DFU group than the NFU group. Sriyani et al. also found that illiteracy was a predictor of increased risk for DFUs in their study conducted in Sri Lanka [4]. This could be explained by the fact that people who are less educated tend to be less aware and have inadequate knowledge regarding health-related issues such as warning signs and regular foot inspection [14, 15]. However, our study also showed that the DFU group had a higher percentage of patients with university diplomas compared to the NFU group. This seems contradictory, but may be related to employment, as the majority (72%) of patients with university diplomas were employed and the DFU group in our study had significantly higher proportion of employed and lower proportion of unemployed patients compared to the NFU group. Employed people are generally busier and have less time to take care of their feet compared to unemployed people. Our study found that diabetic patients with foot ulcers had significantly less monthly income than diabetic patients without foot ulcers. This could be explained in that patients with a lower income have fewer opportunities for healthcare services and are less likely to have appropriate therapeutic footwear. This finding is also supported by Sriyani et al. who found that low income was associated with an increase in the risk of foot ulcer development [4].

Additionally, there were significant differences between patients with and without foot ulcers in terms of type of medication, with the percentage of patients who take insulin higher in the DFU group (39.4%) than that in the NFU group (28.8%). Insulin use is associated with poor diabetic control, which can also be considered a predictor of the development of foot ulcers. Hu et al. also found that insulin use was a predictor for DFUs in their study of 598 diabetic patients in Saudi Arabia [7] as did Yazdanpanah et al. in their study of incidence and risk factors of diabetic foot ulcers in Iran [16]. Our study found that the mean duration of diabetes in years was significantly higher in the DFU group compared to that in the NFU group. This was also a finding of Deribe et al. in their cross-sectional study of 216 diabetic patients in Ethiopia [5]. Two studies in Saudi Arabia have also found a correlation between longer duration of diabetes and development of DFUs [7, 10]. The association of a longer duration of diabetes with DFUs is expected: the longer the duration of diabetes, the higher are the risks for occurrence of complications such as foot ulcers. Also, our study found that the frequency of patients with HbA1c > 7 was significantly higher in the DFU group compared to that in the NFU group (95.2% vs. 62.4%). This result is in agreement with a previous study which found that poor glycemic control increased the likelihood of FU development [10].

Based on the odds ratio analysis, our study showed several independent risk factors for DFU development, including smoking (current and ex-smokers), sensory loss to vibration, sensory loss to monofilament, loss of pedal pulse, and presence of calluses. The study by Sriyani et al. conducted in Sri Lanka also found sensory loss to vibration and abnormal monofilament test results (on the first, third, and fifth toes) as risk factors for DFUs [4].

Also, chronic complications were significantly higher among DFU diabetic patients, including foot deformity, retinopathy, nephropathy, and neuropathy. This could be explained by the fact that diabetes leads to microangiopathic changes [17]. Our findings are consistent with previous studies [8, 10, 16, 18]. Kidney dysfunctions can lead to a delay in the wound healing process and can contribute to the development of foot lesions/ulcers [19]. The presence of retinopathy in DFU patients, which is associated with a decrease in vision, increases the likelihood of foot trauma [20]. This can be explained by a previous study done by Salameh et al. that retinopathy is the most common complication among diabetic patients in Palestine [21]. According to a cohort study in Saudi Arabia with 605 patients with a history of DFU or amputation, gender, distal neuropathy, and foot deformity were independent risk factors for developing DFU [10].

In regard to self-care behaviors, the results in our study revealed that the mean scores of the DFSBS were significantly higher for the NFU group compared to the DFU group. This positive association was also found in previous studies between the development of FU and inadequate foot self-care. A cross-sectional study on prevalence, knowledge, and self-care practices related to diabetic foot among diabetic patients conducted by Chiwanga et al. in Tanzania found that poor self-care behaviors were related to development of FUs [22]. A study of 131 Korean diabetics found that moderate self-care behaviors were demonstrated among DFU patients [23]. Having the ability to perform suitable foot care has been proposed to be positively affected by patient persistent training and knowledge, which can decrease the risk of foot ulceration [24]. Ahmed et al. also found that inappropriate footwear is a risk factor for developing ulceration for diabetics in Saudi Arabia [8]. However, a longitudinal study on 295 diabetic patients in northern Taiwan conducted to determine the effect of certain self-care behaviors on the development of diabetic foot ulcers concluded that self-care behaviors were not sufficient enough to prevent the development of diabetic foot ulcers in patients with neuropathy [9]. Promotion of self-care behaviors among diabetics should have positive results, but other risk factors mentioned above should not be disregarded.

## 5. Limitations

Firstly, the data were only collected from diabetic public primary healthcare centers, which did not take into consideration private diabetic clinics. Secondly, chronic complications were collected retrospectively from the patients' existing medical files. Moreover, the role of family support and diabetes distress as a predictor for foot self-care behaviors and its association with FU development were not accounted for in our research, which warrants further study.

Due to the lack of equipment and specialists available in Palestine, a complete vascular screening including palpation of distal pulses, ankle-brachial index, toe-brachial index, and tcpO2 was not undertaken with study participants. Future studies should include this aspect in case that vascular specialists and necessary equipment become available.

## 6. Conclusion and Implications for Practice

This study showed several independent risk factors of DFU development, which were smoking, sensory loss to vibration, sensory loss to monofilament, loss of pedal pulse, presence of calluses, nephropathy, retinopathy, and neuropathy. Also, this study has shown that illiteracy and lower income were significantly associated with DFU development. Moreover, the current study demonstrated that poor self-care behaviors were associated with DFU. Few studies in Palestine have been done to study diabetic foot ulcers. This research can enable healthcare professionals to better understand risk factors for FU development. The specific factors that contribute to diabetic foot complications may now be taken into consideration. Knowing the predictors and related risk factors will help physicians and nurses to design appropriate programs fitted to reduce the incidence of FU development, such as integrating audio-visual teaching strategies. Also, since many of the risk factors are modifiable, if the healthcare team can increase public awareness and knowledge about this problem and the importance of self-care practices, this could contribute to lowering the incidence of diabetic foot ulcers in Palestine. The information gained from the study will contribute to the education of nursing students in Palestine regarding diabetic foot prevention programs in the community during clinical practice.

## Figures and Tables

**Table 1:** Items in the DFSBS ([13]).

Item	Description
A1	I (my caregiver) examine the bottoms of my feet
A2	I (my caregiver) examine between the toes of my feet
A3	I (my caregiver) wash between my toes
A4	I (my caregiver) dry between my toes after washing
B2	If my skin is dry, I (my caregiver) apply moisturizing lotion to my feet
B7	Before I put on my shoes, I (my caregiver) check the inside of the shoes
B9	I break in new shoes slowly

DFSBS = diabetes foot self-care behavior scale.

**Table 2:** Basic characteristics of participants and comparison between DFU (diabetic patients with foot ulceration) and NFU (diabetic patients without foot ulceration) groups.

Variable	Total (*n* = 413)	NFU (*n* = 205)	DFU (*n* = 208)	*P* value
Age (in years)^∗^	58.2 ± 13.3	57.9 ± 12.6	58.5 ± 13.9	0.633

Gender				*0.378*
Male	206 (49.9)	107 (52.2)	99 (47.6)	
Female	207 (50.1)	98 (47.8)	109 (52.4)	

Region				*<0.001*
South	68 (16.5)	4 (2.0)	64 (30.8)	
Center	61 (14.8)	0 (0)	61 (14.8)	
North	284 (68.8)	201 (98.0)	83 (39.9)	
Income, NIS^∗^	2395.9 ± 1110.1	2600.7 ± 1203.9	2194.0 ± 970.5	*<0.001*

Marital status				*0.598*
Single	39 (9.4)	20 (9.8)	19 (9.1)	
Married	292 (70.7)	145 (70.7)	147 (70.7)	
Divorced	22 (5.3)	8 (3.9)	14 (6.7)	
Widow	60 (14.5)	32 (15.6)	28 (13.5)	

Educational level				*<0.001*
Illiterate	68 (16.5)	19 (9.3)	49 (23.6)	
Primary school	114 (27.6)	63 (30.7)	51 (24.5)	
Secondary school	1113 (27.4)	71 (34.6)	42 (20.2)	
University	118 (28.6)	52 (25.4)	66 (31.7)	

Employment				*0.014*
Unemployed	218 (52.8)	121 (59.0)	97 (46.6)	
Employed	195 (47.2)	84 (41.0)	111 (53.4)	

Type of diabetes				*0.478*
Type 1	34 (8.2)	19 (9.3)	15 (7.2)	
Type 2	379 (91.8)	186 (90.7)	193 (92.8)	

Type of medication				*0.024*
Tablets	259 (62.7)	137 (66.8)	122 (58.7)	
Insulin	141 (34.1)	59 (28.8)	82 (39.4)	
Both	13 (3.1)	9 (4.4)	4 (1.9)	

Amount of insulin				*0.179*
0 units	259 (62.7)	137 (52.9)	122 (58.7)	
<20 units	12 (2.9)	3 (1.5)	9 (4.3)	
20-40 units	66 (16)	31 (15.1)	35 (16.8)	
>40 units	76 (18.4)	34 (16.6)	42 (20.2)	
Fasting blood sugar^∗^	159.1 ± 57.4	156.6 ± 56.5	161.6 ± 58.2	*0.380*

HbA1c				*<0.001*
≤7.0	87 (21.0)	77 (37.6)	10 (4.8)	
>7.0	346 (79.0)	128 (62.4)	198 (95.2)	

Duration of diabetes (in years)^∗^	11.2 ± 7.3	10.1 ± 7.2	12.3 ± 7.3	*0.002*
BMI∗	30.8 ± 5.4	30.7 ± 5.3	30.9 ± 5.5	*0.687*

∗These variables are described as mean ± standard deviation; all other variables are presented as *N* (%) within each group. NIS = New Israeli Sheqel.

**Table 3:** Comparison of risk factors between DFU (diabetic patients with foot ulceration) and NFU (diabetic patients without foot ulceration).

Variable	Total (*n* = 413)	NFU (*n* = 205)	DFU (*n* = 208)	Odds ratio (95% CI)	*P* value
	*N* (%)				
Smoking (current or ex-smoker)	193 (41.2)	80 (39.0)	113 (54.3)	1.86 (1.26, 2.75)	*<0.001*
Hypertension	250 (60.5)	139 (67.8)	111 (53.4)	0.54 (0.36, 0.81)	*0.003*
Sensory loss to vibration	128 (31.0)	50 (24.4)	78 (37.5)	1.86 (1.22, 2.84)	*0.004*
Sensory loss to monofilament	167 (40.4)	64 (38.3)	103 (49.5)	2.16 (1.45, 3.23)	*<0.001*
Loss of pedal pulse	48 (11.6)	15 (7.3)	33 (15.9)	2.39 (1.25, 4.54)	*0.009*
Callus of the feet	200 (48.4)	82 (41.0)	118 (56.7)	1.97 (1.33, 2.91)	*0.001*
Foot deformity	150 (36.3)	53 (25.9)	97 (46.6)	2.51 (1.66, 3.79)	*<0.001*
Dry/cracked foot skin	249 (60.3)	131 (63.9)	118 (56.7)	0.74 (0.50, 1.10)	*0.159*
Retinopathy	182 (44.1)	70 (34.1)	112 (53.8)	2.25 (1.51, 3.34)	*<0.001*
Nephropathy	103 (24.9)	27 (13.2)	76 (36.5)	3.80 (2.31, 6.21)	*<0.001*
Neuropathy	304 (73.6)	132 (64.4)	172 (82.7)	2.65 (1.67, 4.18)	*<0.001*

**Table 4:** DFSBS (diabetes foot self-care behavior scale) scores in diabetic patients and comparison between DFU (diabetic patients with foot ulceration) and NFU (diabetic patients without foot ulceration) groups.

Variable^1^	Total (*n* = 413)	NFU (*n* = 205)	DFU (*n* = 208)	*P* value
	Mean ± SD			
A subscale score	12.70 ± 4.78	13.63 ± 4.77	11.78 ± 4.61	*<0.001*
B subscale score	6.16 ± 2.80	6.62 ± 3.21	5.70 ± 2.23	*<0.001*
Total score	18.86 ± 6.65	20.26 ± 7.18	17.49 ± 5.79	*<0.001*

^1^The A subscale is the total of the scores of the items A1 to A4 (A1: I (my caregiver) examine the bottoms of my feet; A2: I (my caregiver) examine between the toes of my feet; A3: I (my caregiver) wash between my toes; A4: I (my caregiver) dry between my toes after washing). The B subscale is the total of the scores of the items B3, B7, and B9 in the DFSBS (B3: if my skin is dry, I (my caregiver) apply moisturizing lotion to my feet; B7: before I put on my shoes, I (my caregiver) check the inside of the shoes; B9: I break in new shoes slowly). SD = standard deviation.

## Data Availability

The data used to support the findings of this study are available from the corresponding author upon request.
